# Loneliness and intersubjectivity: A view from Trevarthen's theory

**DOI:** 10.3389/fpsyg.2023.1145739

**Published:** 2023-03-09

**Authors:** Evangelia Galanaki

**Affiliations:** Department of Primary Education, National and Kapodistrian University of Athens, Athens, Greece

**Keywords:** loneliness, solitude, intersubjectivity, emotion, motive, sharing, culture

## 1. Introduction

Based on his pioneering research on infants and drawing from the epistemological framework of phenomenology (Husserl, 1964; Habermas, 1972), Trevarthen has formulated a developmental theory of *intersubjectivity* which focuses on innate Other-awareness, inborn motives for sympathetic engagement with others, and cultural sharing—from the beginning of life (Trevarthen, 1977, 1980; Trevarthen and Aitken, 2001). I suggest that this theory of companionship has the potential to offer a rich conceptualization of *loneliness*, defined as the distress and social pain stemming from being alone. This potential has not been examined thus far and is the focus of this study.

## 2. Loneliness as a social or relational emotion

I suggest that within Trevarthen's theory of intersubjectivity loneliness can be regarded as a complex and dynamic *social* or *relational emotion* (a term introduced by Stern, 1993). It is an emotion between persons, a fellow-feeling, according to philosopher Adam Smith (1759), who inspired Trevarthen.

Social or relational emotions belong to the *causes* of consciousness, according to Trevarthen (2005a). This may apply to loneliness too and contrasts with the long-standing interpretations of loneliness as an outcome of cognitive processes, that is, of individuals' awareness of their relational (quantitative and/or qualitative) deficits, which, in turn, stems from the perception of the dissonance between the expected and the real level of relationships (e.g., Peplau and Perlman, 1982). Cognitive models of loneliness place emphasis on *subjective* cognitions as the source of loneliness. In the intersubjective framework, the order is reversed: social emotions are the causes of co-consciousness, of self-other awareness.

Loneliness is a universal experience with cognitive, emotional, contextual, and motivational dimensions (e.g., Galanaki, 2004). From an evolutionary and attachment viewpoint, it has been conceptualized as a proximity-promoting mechanism with a survival value for the human species (Cacioppo and Patrick, 2008). However, the theory of intersubjectivity has the potential to place loneliness over and above attachment (protection, comfort, and care) and survival. Thus, loneliness may be regarded as expressing not only attachment for care but also *attachment for companionship* and as arising and being alleviated within a *circle of attachments* (Trevarthen, 2004a, 2005c). In this line of thought, loneliness originates from humans' *social brain* (Dunbar, 2009), *allocentric perception* (Bråten, 2009), and innate *dialogicity* (Wertsch, 1991).

## 3. Loneliness as a moral emotion

Further, I suggest that, as a motive for *sympathy*, loneliness belongs to our moral core and can be regarded as a *moral emotion*. Unlike empathy, which is rather one-sided, sympathy is the intersubjective awareness of agency and emotion that works reciprocally between persons (Reddy and Trevarthen, 2004; Reddy, 2008) and reflects complementary emotional states that are shared with the other. It is a bridge between persons expressing their mutual assistance (Trevarthen, 2013). Sympathetic persons have moral emotions, such as pride and shame, that “can keep or break social ties, and may facilitate sharing of meanings and purposes, or make their understanding more difficult” (Gratier and Trevarthen, 2007, p. 173). Specifically, Trevarthen (2005c, p. 77) links shame and loneliness by stating that “shame in failure […] threatens loss of relationship and hopeless isolation.”

Aloneness carries stigma and signifies rejection, exclusion, and ostracism. This is often an *exclusion from meaning* (Trevarthen, 2005c). Therapists must keep in mind that we are born with readiness to join in kindness and playful sympathy with companions (Trevarthen, 2019). Thus, *loneliness of shame* (Trevarthen, 2022) may be the target of prevention and therapy from the beginning of life.

## 4. Loneliness as an innate intersubjective motive

If emotions and motives have strong links with each other or even overlap (Trevarthen, 1993), I suggest that loneliness can also be conceptualized as an *innate intersubjective motive*—a motive for seeking human company. Loneliness stems from absence and is regarded as longing for something missing. The desire to transform absence into presence—the *compulsion to share* the time of movement (Trevarthen, 2009)—may be what distinguishes loneliness from clinical depression (however, intense loneliness may appear as a symptom of clinical depression and take on several qualities as a function of the type and severity of depression). In depression, motives for life and companionship as well as hope for the future seem to suffer. Therefore, loneliness is a *measure of companionship*, not separateness (Trevarthen, 1998). It is “foundational in developing human relations, and in the growth of a sense of individuality or identity in society”—this is Trevarthen (2002, p. 175) view for the emotions of pride and shame.

If emotions are *reflective*, “in the sense that their usefulness for each individual depends on what emotional signals come back from other individuals” (Trevarthen and Aitken, 2003, p. 9) and we can mirror each other's emotions, a person's loneliness *move others*. All emotions can evoke responses in sympathetic others (Reddy and Trevarthen, 2004). Therefore, I suggest that one's loneliness may be painful for all sympathetic others too but also that it is more tolerable by the lonely person who can share it. Furthermore, loneliness in one person can change the emotional state of the other (the sympathetic) person. Nevertheless, the phrase “in one person” is not accurate, because loneliness floats in-between persons, even though it is felt as a highly private experience.

If motives are *observable* (Trevarthen, 1998), it is possible (although not always easy) to detect when others are lonely and seek company. And if motives are transferred and used co-operatively, we can be invited by others to enter their loneliness and contribute to its alleviation.

## 5. The origins of loneliness and solitude

Regarding loneliness, if infants have feelings like ours (Trevarthen, 2005a), they are bound to have this experience, although they cannot use language to convey it. This may be one reason why their loneliness is neglected by researchers. Trevarthen (2005a, p. 62) wonders: “What causes an infant to display rage or sad withdrawal in a relationship that is not working as expected, and why does a contented infant's mind hide behind a silent mask of inwardness, apparently inventing thoughts?” A fear of an unsupported loneliness is considered typical of the newborn child (Trevarthen, 2003). A baby can express sadness when alone and in need (Trevarthen, 2005b, 2015) and loneliness is one of the outcomes of insensitive, neglectful, or intrusive parenting (Trevarthen, 2014). Trevarthen does not place much emphasis on infants' separation anxiety, which has been regarded as fear of loneliness (Bowlby, 1973; Quinodoz, 1993), perhaps because of his critical attitude toward attachment theory (Trevarthen, 2005c).

Furthermore, solitude is an experience related to loneliness, yet also distinct from it. It is usually conceptualized as *time alone* and as a *state of mind*, rather than a state of being [i.e., it may or may not include physical separation (Coplan et al., 2021)] and is described as a *paradox* because, although it can be self-enhancing, it may lead to loneliness (Galanaki, 2005, 2021). Although Trevarthen (2004b) stresses that there are no single infant heads, he states that even very young infants are capable of disengagement and detachment from sharing of impulses and feelings with other human beings and often withdraw into a solitary state of thinking, reflection, and *contemplation* (Trevarthen, 2011a). It is a state of *self-synchrony* which includes body movements, facial expressions, and vocalizations (Trevarthen, 2011a) and reflects a third type of *motive* (the other two are communicating with persons and doing with objects; Trevarthen, 1998). This inward or self-directed motive has been neglected by infant research (Trevarthen, 1998). Private thinking and social communicating co-exist in corresponding and complementary ways from the beginning of life. The minds of mother and infant are together while having their separate recollections and purposes and while sharing these reflective, meditative states (Hobson, 2002; Trevarthen, 2005a). I would call this shared experience of mother and infant *solitude à deux* and I suggest that, if sympathy means *respect* for the other person's autonomy even when there is disapproval (Trevarthen, 2005c), it is the sympathetic mother that sets the stage for her child's life-long capacity to benefit from solitude. The mother's respect for her child's autonomous ingenuity and invention—this respect is also an educational value (Trevarthen, 2011b)—echoes (Winnicott's, 1965) statement that the *capacity to be alone in the presence of the mother* is a major developmental achievement.

## 6. Implications for the causes and the alleviation of loneliness

Trevarthen's research and theory of intersubjectivity have the potential to offer a deeper understanding of the causes and, therefore, the alleviation of loneliness. Throughout life loneliness results from a *failure in intersubjectivity* and is reduced when *meaningful sharing* is restored and maintained.

More specifically, first, loneliness may arise when one is *literally* alone. Then, real time engagement with a partner in a dialogue is not possible. The needs for companionship, sharing of vitality, joy, and pleasure are not satisfied. The *actual Other* is missing and the *implicit* or *virtual Other* (Bråten, 1992) is inadequate or the actual Other is missing for a long period. Within the epistemological framework of phenomenology (e.g., Heidegger's being-with-others, Husserl's transcendental intersubjectivity, and Merleau-Ponty's intercorporeity), prolonged and imposed solitude, as a privation of primary and secondary intersubjectivity, throughout life, is a severe *existential* disruption, because it undermines our very constitution—the relational self—and, thus, leads to disturbances in the sense of realness (Gallagher, 2014).

Second, loneliness emerges from the imperfections or distortions of *co-regulation* with partners, from lack of harmony. Trevarthen often uses the terms *mirroring* (Winnicott, 1971) and *attunement* (Stern, 2000) to describe this co-regulation. “Reading” the Other and being “read” by the Other is impaired in loneliness. Our initiative, as an invitation and provocation of the Other, is not reciprocated. Our anticipation is frustrated and our offerings (initially, as research has shown, in imitation and proto-conversation; Murray and Trevarthen, 1985; Kugiumutzakis et al., 2005; Kugiumutzakis and Trevarthen, 2015) are not acknowledged. No loving voice is heard or there is no one to hear our voice (perhaps because of mother's own sense of loneliness and not belonging; Gratier et al., 2015) and to reciprocate our gaze and touch. There is a failure to participate in shared time (Trevarthen, 2016). *Synrhythmia*—the Greek term that Trevarthen et al. (2006) used to capture the graceful poly-rhythmic resonance with the Other and expresses our innate communicative musicality (Malloch and Trevarthen, 2009)—is lost.

Third, I suggest that *distortions of sharing* may be mutually related to loneliness. Optimal sharing of emotions, motives, interests, purposes, actions, etc. is regarded to reflect a balance between engagement and disengagement. When disengagement is more or less than desired, loneliness emerges. These difficulties in self- and co-regulation may manifest as conflicts regarding whether to share or not to share; what to share and what not; when, where, in what pace and with whom to share. In other instances, sharing is asymmetrical or the partners' expectations for the amount of sharing do not match, or sharing is excessive (i.e., one feels “transparent” in an encounter). When chronic and intense, loneliness, in turn, may lead to distortions of sharing. For example, in less fortunate cases, the motivational force of loneliness is not so strong as to facilitate the sharing of our loneliness story with others or, even more, the sharing of the *illusion of sharing* (Kugiumutzakis, 2012; see also Reddy, 2008).

Fourth, because of the distortions of sharing, we cannot co-create *meaning*. Acts of meaning (Bruner, 1990) are impeded or discouraged. Meaning is created when we participate in *emotional narratives* with the Other (starting early in infancy; Stern, 2000) and these embodied narratives gradually become social schemas with cultural significance (Delafield-Butt, 2018). Trevarthen (2004b, p. 23) states that the search for meaning “can fall prey to fear and distress, loneliness and self-doubt.” From the beginning of life, we crave for reliable and affectionate others who can sustain the memories we created with them. Sometimes, however, we cannot draw from treasured memories of a special relationship (Trevarthen, 2008), perhaps because there are no such memories, or we cannot sustain the co-discovered memories. In loneliness, we are not meaningful to a significant Other (initially, less fortunate infants are not meaningful to their mothers in the mother-infant proto-conversation).

Finally, apart from the presence of an actual Other, synrhythmia, sharing and co-construction of meaning, it is *cultural membership*, that is, finding one's place in the world as a doer and knower (Trevarthen, 2004b), that reduces loneliness. In the cultural context (initially, the mini-culture of the mother-infant dyad), we satisfy our social curiosity by sharing mental spaces and thoughtfulness. We find sympathetic and trusted partners to share our stories and our sense of beauty (Trevarthen and Delafield-Butt, 2017). “Loneliness, shame, depression and sadness are the emotions that identify loss of this *collective story-telling*, which can be called ‘socionoesis”' (Trevarthen, 2013, p. 204; see also Delafield-Butt and Trevarthen, 2015). But if we manage to co-create a narrative about cosmos and feel our co-existence in *symbolic* and *collaborative awareness* (Trevarthen et al., 2006) and our contribution (doing and knowing) is valued by others, *cultural learning* is facilitated and *pride in meaning* (Trevarthen, 2001), instead of shame, is felt. Trevarthen (2004b, p. 36) suggests that “all human cultural achievements arise shared meanings, even when they appear to be lonely products, of creatively dreaming or of adventurous risk-taking in thought or action”. To conclude, loneliness arises in a community of minds and is moderated by cultural membership and cultural sharing.

## Author contributions

The author confirms being the sole contributor of this work and has approved it for publication.
